# Study of an Oxygen Supply and Oxygen Saturation Monitoring System for Radiation Therapy Associated with the Active Breathing Coordinator

**DOI:** 10.1038/s41598-018-19576-8

**Published:** 2018-01-19

**Authors:** Guanzhong Gong, Yujie Guo, Xuemei Sun, Xiuying Wang, Yong Yin, David Dagan Feng

**Affiliations:** 1 The Radiation Oncology Department of Shandong Cancer Hospital, Affiliated To Shandong University, Jiyan Road 440#, Jinan Shandong, 250117 China; 2 Biomedical And Multimedia Information Technology (BMIT) Research Group, School Of Information Technologies (SIT), The University Of Sydney, Sydney, Nsw 2008 Australia; 3 The Intensive Care Unit Of Shandong Cancer Hospital, Affiliated To Shandong University, Jiyan Road 440#, Jinan Shandong, China 250117

## Abstract

In this study, we designed an oxygen supply and oxygen saturation monitoring (OSOSM) system. This OSOSM system can provide a continuous supply of oxygen and monitor the peripheral capillary oxygen saturation (SpO2) of patients who accept radiotherapy and use an active breathing coordinator (ABC). A clinical test with 27 volunteers was conducted. The volunteers were divided into two groups based on the tendency of SpO2 decline in breath-holding without the OSOSM system: group A (12 cases) showed a decline in SpO2 of less than 2%, whereas the decline in SpO2 in group B (15 cases) was greater than 2% and reached up to 6% in some cases. The SpO2 of most volunteers declined during rest. The breath-holding time of group A without the OSOSM system was significantly longer than that of group B (p < 0.05) and was extended with the OSOSM system by 26.6% and 27.85% in groups A and B, respectively. The SpO2 recovery time was reduced by 36.1%, and the total rest time was reduced by 27.6% for all volunteers using the OSOSM system. In summary, SpO2 declines during breath-holding and rest time cannot be ignored while applying an ABC. This OSOSM system offers a simple and effective way to monitor SpO2 variation and overcome SpO2 decline, thereby lengthening breath-holding time and shortening rest time.

## Introduction

Breathing motion is a critical factor that affects the accuracy of radiotherapy for thoracic and abdominal cancers; it can induce errors in tumor localization, treatment planning design, patient setup and dose delivery^1^. These errors can result in uncertain doses of radiation to the target tumor volume and organs at risk (OARs), which increases the risks of treatment failure and of radiation injury to OARs. Thus, it is important to solve problems involving breathing motion in the context of thoracoabdominal tumor radiation therapy. Breathing motion management methodologies (e.g., active breathing control, breath gating, and abdominal compression) have played important roles in decreasing the motion effect on radiotherapy precision^2^.

*Active breathing coordinator*^*TM*^ (ABC; *Elekta Oncology, Crawley, UK)* is an active breathing control technology that helps patients achieve and maintain their breath-holding status for up to 30 s or even longer through a special pipe. Theoretically, the ABC can eliminate the errors affecting tumor target volume position and the dose delivery induced by the breathing motion, and it has been applied at our radiation oncology clinic for more than 18 years since it was first reported by Wong *et al*. in 1999^3^. Furthermore, encouraging dosimetric benefits have been obtained via the precise radiation therapy of non-small cell lung cancer (NSCLC), breast cancer, lymphoma and liver cancer^4–8^. Our preliminary work proposed a method that combined volumetric modulated radiation therapy with active breathing control during moderate deep-inspiration breath-holding. We demonstrated that the mean dose and V20 (i.e., the percentage of the normal lung receiving at least 20 Gy) could be reduced by more than 18% and 30%, respectively, during radiation therapy of thoracic esophageal carcinoma^9^.

The patient’s body is in a temporary non-ventilation state when ABC is used. Moreover, their lung function is affected, which creates uncertainty for the stability and reproducibility of the ABC application^10^. Furthermore, it creates additional concerns with regard to high dose per fraction radiation therapy modalities, such as stereotactic body radiation therapy (SBRT), which can reach up to 12 Gy/fraction or more among patients with lung cancer who need more time for every irradiation beam and fraction without an objective indication with monitoring indices^11^. Zhong *et al*. reported that the breath-holding time extended to over 40 s with the help of inhaling oxygen during SBRT of liver cancer with ABC. However, research addressing the physiological functional changes in long breath-holding time is rarely reported^12^. Therefore, it is difficult for radiation therapists to evaluate the human physiologic condition; on occasion, the choice of moments of beam-on and beam-off and that of the length of breath holdingtime and rest time are blind. The methodologies used to monitor human physiological changes and improve physiological functioning will help the use of ABC. Furthermore, it would assist radiation therapists in making objective decisions regarding the moment of beam-on and beam-off, as well as the start and stop of patient rest.

ABC devices have been used at our hospital for more than a decade (since 2003), and the new ABC, version 2.0 (v.2), was recently utilized at our hospital. We designed an oxygen supply and oxygen saturation monitoring (OSOSM) system and performed a preliminary clinical test. We compared the peripheral capillary oxygen saturation (SpO2), breath-holding time and rest time variations for inhaling or not inhaling oxygen using the OSOSM system. The feasibility of its clinical application for decision making was also studied.

## Materials and Methods

### Monitoring system framework and its implementation

**Figure 1:**
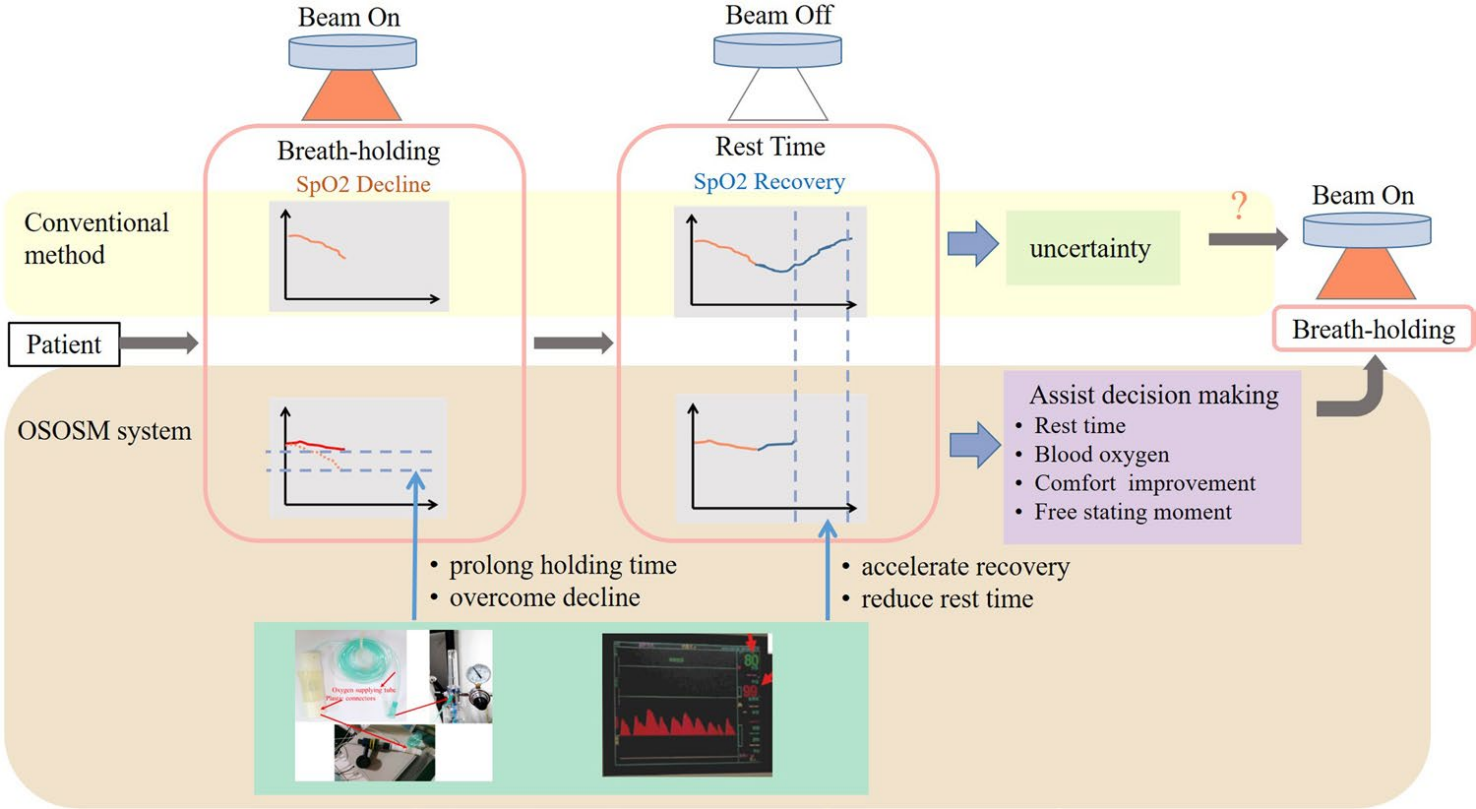
Motivation for and framework of the proposed OSOSM system. The OSOSM system can prolong the breath-holding time, shorten the rest time, overcome SpO2 decline and accelerate SpO2 recovery via the continuous supply of oxygen. Furthermore, real-time SpO2 data may offer an objective index to determine the starting moment of beam-on and beam-off s, as well as the lengths of breath-holding time, single breath-holding time and rest time.

We created an OSOSM system that incorporates SpO2 monitoring and oxygen-supplying devices into the ABC application (see Fig. 1). We hypothesized that the human physiological condition is reflected in SpO2 variation, which can be used as an effective and objective tool to monitor the variation in blood oxygen content. Furthermore, the continuous oxygen supply can keep patients breathing in high-oxygen concentrations to help improve their blood oxygen content during breath-holding. The proposed system has the potential to ameliorate the SpO2 decline rate, speed up SpO2 recovery, and shorten both patient rest and total treatment times.

### The ABC device: OSOSM system information

**Figure 2:**
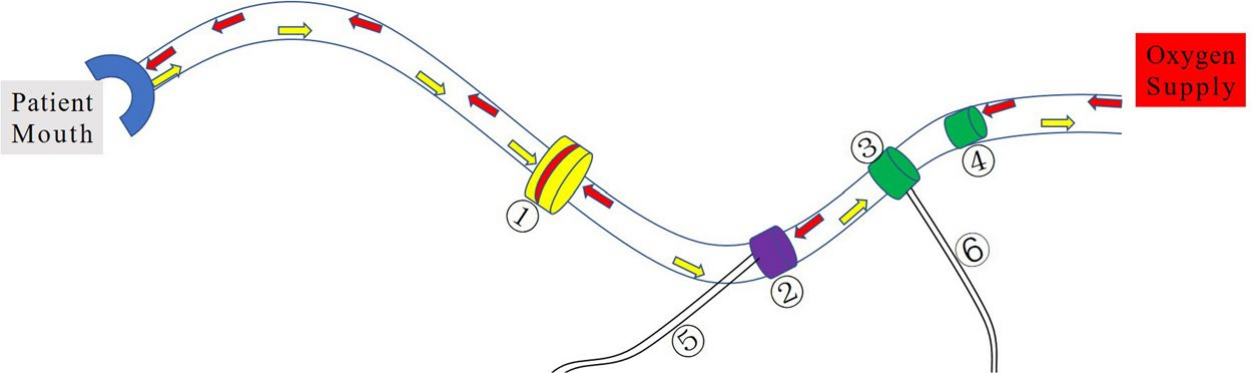
Diagram of the connection pipe and breath-control system of ABC.

In this test, we used the ABC *v.2*, which is able to record and analyze each fraction of the patients’ breathing curves. The connection pipe and breath-control system are shown in Fig. 2. The mouthpiece was used to ventilate by filtering the steam via the filter kit, and the balloon valve was used to help patients achieve a breath-holding motion by inhaling actively at a specific moment.

**Figure 3:**
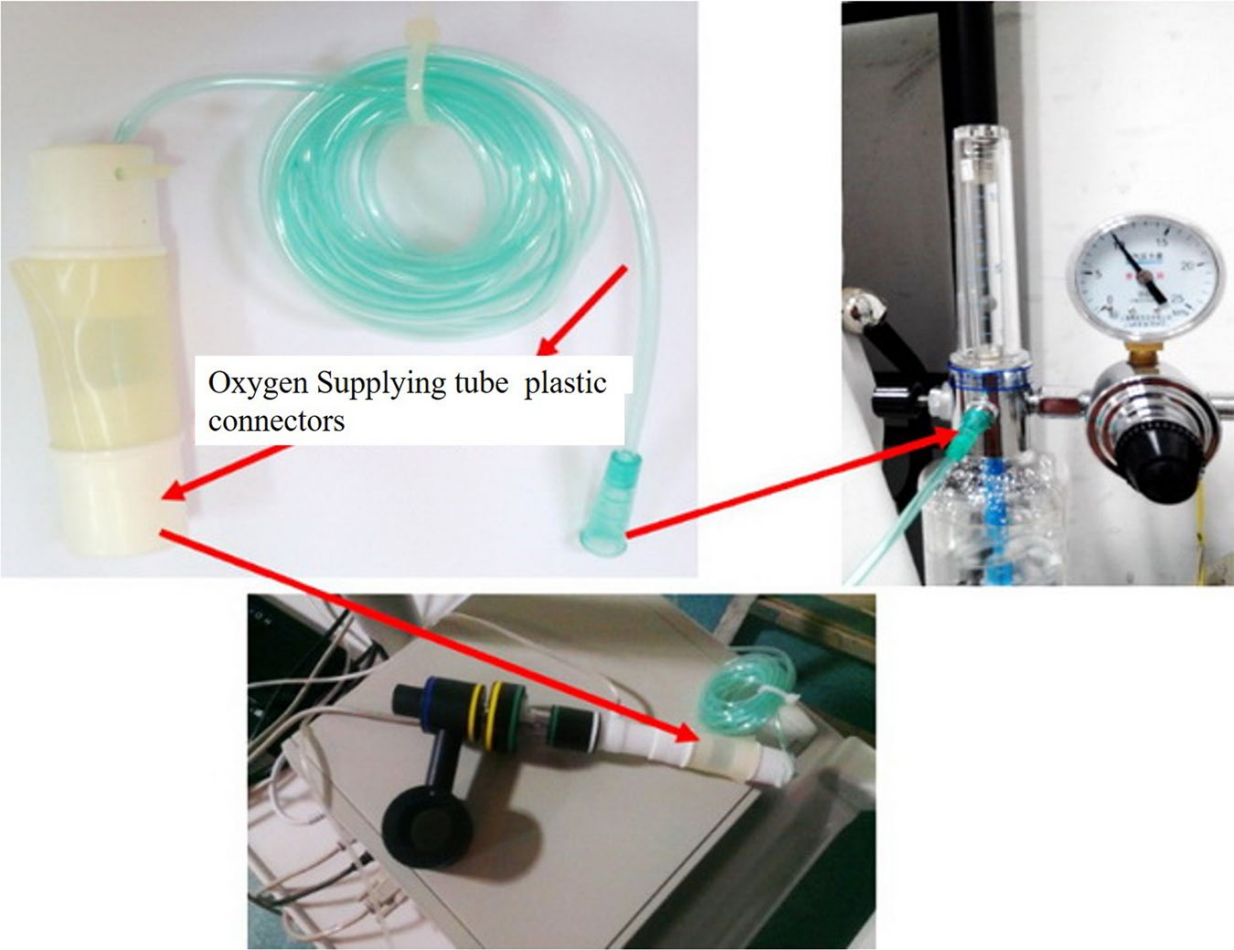
Structure of the extended oxygen pipe and the connection to oxygen bottles.

We also designed an extended oxygen pipe (Fig. 3) that contains plastic connectors and a thin oxygen supply tube. The plastic connectors were used to connect the ABC, whereas the oxygen supply tube was used to continuously deliver oxygen at the speed of 3 L/min from a medical oxygen bottle throughout the ABC application. A PACIFIC PC9000 electrocardiogram monitor with several parameters (SHENZHEN CREATIVE Co., LTD., China) was used to monitor real-time SpO2.

### Clinical test volunteer information

A total of 27 volunteers with normal cardiopulmonary function and the willingness to cooperate were selected for this clinical test (16 men and 11 women, ranging from 22 to 35 years old). The cardiopulmonary functions required were a vital capacity of >2000 ml, a residual volume/total lung capacity of <25 and a forced expiratory volume in one second of >83%; there was no abnormal cardiac function blood index. The Medical Ethics Approval Committee of Shandong Cancer Hospital and Institute reviewed and approved this test, and we confirmed that all the experiments within this study were performed in accordance with the relevant guidelines and regulations. All the volunteers consented to participate in this study and signed a written informed consent document at the time of admission.

### Clinical test protocol

All volunteers were requested to breathe through a mouthpiece connected to an ABC and hold their breath at the end of inspection 10 times with or without inhaling oxygen successively. An electrocardiogram monitor was used to detect the variations in SpO2. The next breath-holding task was not started until the SpO2 had recovered to normal.

**Figure 4:**
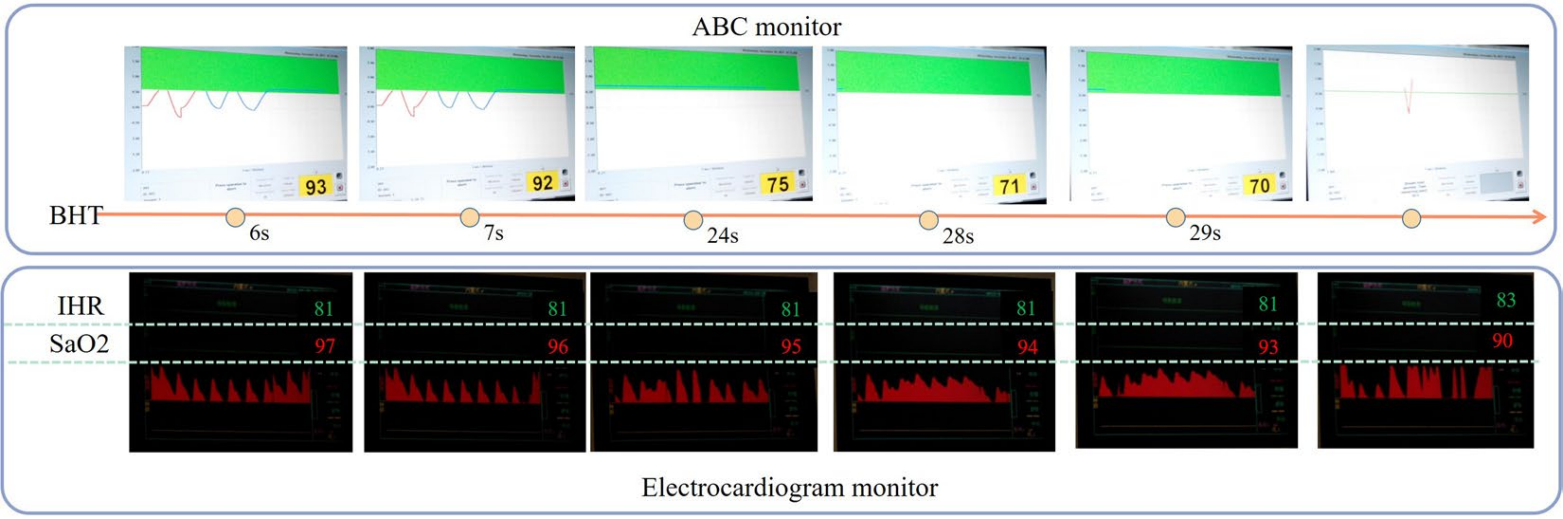
SpO2 variation of one volunteer with or without inhaling oxygen over time.

As shown in Fig. 4, a camera was used to record all survey processes of each volunteer. The SpO2, breath-holding time and rest time experimental data were analyzed using the frames of each video. To reduce the artificial bias induced by random outliers, we did not evaluate the maximum and minimum values of each volunteer. A normal SpO2 range was defined as 95% to 100%.

### Assessment of SpO2

The standard SpO2 decline rate was considered as surpassing 2% over 2 s without rising from the beginning to the end of the breath-holding period.

### Statistical analyses

SPSS 16.0 (IBM, Chicago, IL, USA) was used for all statistical analyses. One-way analysis of variance (ANOVA) was used for data comparison. A difference of *p* < 0.05 was considered as significant. The datasets generated during the current study are available from the corresponding author on reasonable request.

## Results

### Overview of the clinical test results

All 27 volunteers completed the clinical test successfully without any reported incident. A total of 19 volunteers stated that they felt obvious improvements in comfort during the breath-holding period and experienced shortened rest time after inhaling oxygen, whereas the other 8 volunteers reported no significant improvement.

### Group division of the volunteers

**Figure 5:**
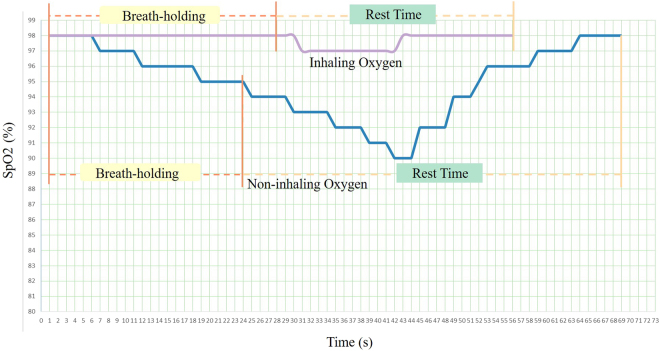
Curve of one volunteer’s SpO2 variations with or without inhaling oxygen.

The volunteers were divided into two groups according to their SpO2 variations in breath-holding without inhaling oxygen. In group A (12 cases), the SpO2 declined less than 2%, whereas in group B (15 cases), SpO2 decline was greater than or equal to 2% and lasted longer than 2 s without rising again (see Fig. 5 and Tables 1 and 2).

**Table 1:** SpO2 comparisons of groups A and B at the beginning and end of breath-holding and the minimum rest time value with or without inhaling oxygen.

		SpO2_-beg_ (%)	SpO2_- end_ (%)	SpO2_-least_ (%)
A	NIO	98.87 ± 0.39	98.63 ± 0.55	98.27 ± 1.16
	IO	99 ± 0	98.87 ± 0.468	98.75 ± 0.73
		*F* = *7.045, p* = *0.01*	*F* = *6.244, p* = *0.01*	*F* = *7.451, p* = *0.01*
B	NIO	98.13 ± 0.89	96.15 ± 1.58	94.31 ± 1.76
	IO	98.89 ± 0.32	98.49 ± 0.66	97.74 ± 1.72
		*F* = *51.95, p* = *0.00*	*F* = *148.72, p* = *0.00*	*F* = *154.88, p* = *0.00*

Note: SpO2_-beg_: SpO2 at the beginning of breath-holding; SpO2_- end_: SpO2 at the end of breath-holding; SpO2_-least_: the minimum SpO2 during rest time; NIO: not inhaling oxygen; IO: inhaling oxygen.

**Table 2:** SpO2 variations of groups A and B during the breath-holding and rest times with or without inhaling oxygen.

		_∆_SO2_-vdbh_ (%)			_∆_SO2_-vdrt_ (%)		
		Max	Min	$\bar{x}$ ± S	Max	Min	$\bar{x}$ ± S
A	NIO	2	0	0.23 ± 0.46	5	0	0.37 ± 0.90
	IO	2	0	0.13 ± 0.46	3	0	0.12 ± 0.49
		*F* = *3.562, p* = *0.06*			*F* = *1.38, p* = *0.243*		
B	NIO	5	0	1.975 ± 1.31	6	0	1.84 ± 1.53
	IO	3	0	0.40 ± 0.63	3	0	0.75 ± 1.73
		*F* = *93.793, p* = *0.00*			*F* = *17.72, p* = *0.00*		

Note: _∆_SO2-vdbh: SpO2 variation during breath-holding; _∆_SO2-vdrt: SpO2 variation during rest; NIO: not inhaling oxygen; IO: inhaling oxygen: S = standard deviation.

### SpO2 variations during breath-holding with and without inhaling oxygen

The average SpO2 of group A at the beginning of the breath-holding period was higher than that of group B by 0.75% before inhaling oxygen; however, the SpO2 values were similar after inhaling oxygen.

The average SpO2 of group B at the end of the breath-holding period increased by 2.43% after inhaling oxygen but increased by only 0.24% in group A. A similar trend toward improvement (by means of 67.6% vs 61.1%) was found with regard to SpO2 variation during rest time for groups A and B, respectively (see Tables 1 and 2).

Moreover, the amplitude of SpO2 decline in breath-holding with and without inhaling oxygen (*p* > 0.05) did not differ significantly among the volunteers in group A. However, it clearly improved in group B after inhaling oxygen, decreasing from an average of 1.98 ± 1.31% to an average of 0.40 ± 0.63% (*p* < 0.05; see Table 2).

### SpO2 variations during rest time with and without inhaling oxygen

SpO2 declines occurred for most of the volunteers during rest time, and the lowest SpO2 values reached 94% in group A and 90% in group B. After inhaling oxygen, the SpO2 decline value of group A exhibited no significant difference, whereas that of group B decreased significantly from 1.84 ± 1.53% to 0.75 ± 1.73%, on average (*p* < 0.05; see Tables 2 and 3).

**Table 3:** Breath-holding time, time needed for SpO2 to recover to normal and rest times of groups A and B with or without inhaling oxygen.

		BHT(s)			RT_-SpO2_(s)			RT(s)		
		Maxi	Mini	$\bar{x}$ ± S	Maxi	Mini	$\bar{x}$ ± S	Maxi	Mini	$\bar{x}$ ± S
A	NIO	45	18	33.77 ± 7.33	48	15	28.3 ± 8.87	41	19	31.07 ± 6.20
	IO	60	30	42.75 ± 65.58	19	10	14.6 ± 2.88	39	17	22.65 ± 3.8
		*F* = *43.19, p* = *0.00*			*F* = *22.149, p* = *0.00*			*F* = *80.239, p* = *0.00*		
B	NIO	46	22	30.51 ± 4.80	46	15	26.87 ± 5.48	51	26	35.08 ± 4.80
	IO	49	25	39.01 ± 4.29	30	5	20.51 ± 5.51	36	18	25.26 ± 4.37
		*F* = *140.25, p* = *0.00*			*F* = *33.38, p* = *0.00*			*F* = *182.374, p* = *0.00*		
A vs B	*NIO*	*F* = *11.47, p* = *0.00*			*F* = *0.72, p* = *0.40*			*F* = *18.478, p* = *0.00*		
A vs B	*IO*	*F* = *11.81, p* = *0.00*			*F* = *10.63, p* = *0.00*			*F* = *13.654, p* = *0.00*		

Note: Max: maximum; Min: minimum; BHT: breath-holding time; RT-SpO2: time needed for SpO2 to recover to normal; RT: rest time; $\bar{x}$ ± S = mean ± standard deviation.

### Breath-holding time with and without inhaling oxygen

The breath-holding time of group A was prolonged from an average of 33.77 s to 42.75 s, with an increase of 26.60%, whereas that of group B was prolonged from 30.51 s to 39.01 s after inhaling oxygen, for an increase of 27.85%. The increasing amplitude of breath-holding time was similar (by means of 8.9 s vs 8.5 s) after inhaling oxygen in the two groups (see Table 3).

### SpO2 recovery and rest time variations with and without inhaling oxygen

The recovery time of SpO2 (from the end of the breath hold to when the SpO2 value recovered to baseline) in group B was reduced from 26.87 s to 20.51 s, on average, after inhaling oxygen (*p* < 0.05), whereas the rest time was reduced from a mean of 35.08 s to a mean of 25.26 s (*p* < 0.05). In group A, the time needed for SpO2 to recover to normal was reduced from 28.3 s to 14.6 s, and the rest time was significantly reduced from 31.07 s to 22.65 s, on average (*p* < 0.05; see Table 3).

## Discussion

In the present study, an OSOSM system was designed by using simple devices that can be easily found at every hospital. No mechanical collision risk existed among the linear accelerator gantry, table, ABC device and OSOSM system because the long gas-supply pipeline and signal-conducting wire were used to connect the oxygen supply tube and the electrocardiogram monitor to the patients. In the future, an integrated ABC device with the system described above will be manufactured. The electrocardiogram monitoring system is also a proven and valuable non-invasive screening tool to monitor SpO2^13^ SpO2 is an objective indicator of oxygen changes in blood flow; therefore, its decline reflects a reduced oxygen supply to the patient and his or her tumor^14–16^.

There are some specific details which can seriously worsen normal respiratory function in usage of ABC. First, the pipe that connects patients with the ABC device is more than 50 cm in length and holds over 200 ml volume of air, which significantly increases the physiological dead space of the patient’s respiratory system. Second, patient respiratory airway resistance is increased significantly because of the pipe filter kit connection. Furthermore, the increases in airway resistance become even more obvious when the pipe is wet (Fig. 1). Lastly, patients feel pharyngeal discomfort because they must be ventilated via a connecting pipe through the mouth. These factors result in a potential hypoxic state that can affect patient cooperation and tolerance for the ABC. However, the lung function changes associated with the ABC remain unknown at the current stage^17,18^.

Healthy lung and heart function are known prerequisites for ABC application; however, knowledge regarding the variations in SpO2 during breath-holding and its effect on precision radiotherapy is limited^19^. The findings regarding SpO2 variations revealed by our clinical test are very interesting. SpO2 decline occurred in approximately 57.14% of the volunteers during the breath-holding process and in almost 100% of volunteers during rest; however, the volunteers in our study were young and healthy. Furthermore, the finding that the breath-holding time of group B was briefer but the rest time was longer than group A demonstrates that SpO2 decline affects the total time of radiation therapy associated with ABC. Thus, the implementation of this procedure within radiotherapy would be affected, especially for patients with lung cancer or others with thoracic carcinomas with or without surgery.

It has been known that hypoxic tumors showed serious resistance to radiotherapy^20,21^. Hyperbaric inspiratory hyperoxia is one effective approach to improve the hypoxia status of the tumor, although it is difficult to perform irradiation under hyperbaric conditions^22,23^. In our study, the human hypoxia status was effectively improved by inhaling oxygen continuously. This method may successfully increase the oxygen supply to the tumor and its surrounding microenvironment^24,25^. The SpO2 decline of most volunteers during rest may be explained by the changes in blood SpO2 that could delay the actual physiological level of the normal tissues because of the oxygen and carbon dioxide diffusion times. In contrast, the current study suggests that full consideration should be given to SpO2 changes during radiotherapy with application of the ABC. Patients must be given sufficient rest time to improve the oxygen status of their tissues and tumors before the next beam. Our results demonstrate that the mean rest time was over 20 s and that the length of rest time will increase significantly without inhaling oxygen.

The average SpO2 recovery time among the volunteers in group B was reduced by 23% and the average rest time was reduced by 30%; in group A, these values were reduced by 50% and 31.17%, respectively. It is useful to reduce the total treatment time of single-fraction radiation therapy. Over 70% of the volunteers stated that their comfort improved after inhaling oxygen; this improvement was attributed to the rapid increase in blood oxygen concentration. Our results also revealed that although the breath-holding time was prolonged after inhaling oxygen, its intra-fraction variation was reduced, which may have been more important for group B volunteers than group A volunteers. So we recommend that oxygen inhalation should be provided routinely to patients who need radiotherapy associated with ABC because of the promising benefits.

Conventionally, the starting moment of beam-on and beam-off, as well as the length of rest time among the different beams, cannot be easily decided upon without objective quantitative indices^26^. Our monitoring data demonstrated that the SpO2 level cannot be neglected when making decisions about when to stop resting, start the next beam-on treatment, or determine the length of the single breath-holding time. SpO2 must recover to normal before the next beam is turned on, which can reveal that the human was not in potential hypoxic state. It is important to ensure the safety and effectiveness for patients who accept a high fraction dose SBRT associated with ABC^26^. Similar and encouraging results may be obtained by clinical studies of patients with thoracic or abdominal cancer who must accept SBRT in the future; the clinical application of this research is in progress.

The main limitation of this study was that the trial data were obtained from young, healthy volunteers. More encouraging outcomes may have been determined from patients with thoracic or abdominal tumors comorbid with pulmonary dysfunction or deficiency. The major challenge was selecting appropriate patients who would obtain the maximum benefit from using the OSOSM system. Moreover, the cardiopulmonary functions of patients should have been tested to filter the sample. In addition, the patients could have inhaled oxygen for more than 10 minutes before accessing the therapy room, which may have led to better outcomes because of oxygen enrichment.

Radiotherapy with ABC has been performed in our hospital for nearly 15 years. Although the ABC device plays an important role in dose escalation for tumors and dose decreases for OARs^8,9,27^, there are some details that should be given special consideration. Patients should be provided with sufficient time to rest; radiation therapist should choose the right moments to stop rest and start beam-on treatment; the normal shape of the connection pipe should be maintained; the connection pipe should be kept clean and dry (especially the filter); and the tightness of the pipeline should be checked and confirmed^8,9^.

## Conclusions

The clinical test of our OSOSM system demonstrated that the SpO2 decline associated with using ABC cannot be ignored. Furthermore, inhaling oxygen can significantly prolong patient breath-holding time and limit the SpO2 decline while shortening SpO2 recovery and rest time after the ABC is applied. The proposed OSOSM system offers a practical way to improve the use of the ABC via continuous monitoring of SpO2 and the oxygen supply. This system serves as an objective, quantitative index for deciding the optimal moment to start beam-on treatment, raising the SpO2 and shortening the rest time. Moreover, this system holds the potential to improve the efficiency and clinical outcomes of applying the ABC.
